# Impact of dehydration on laparoscopic performance: a prospective, open-label, randomized cross-over trial

**DOI:** 10.1007/s00464-023-10644-3

**Published:** 2023-12-26

**Authors:** Jean-Paul Bereuter, Mark Enrik Geissler, Rona Geissler, Sofia Schmidt, Nathalie Buck, Juliane Weiß, Grit Krause-Jüttler, Jürgen Weitz, Marius Distler, Felix von Bechtolsheim, Florian Oehme

**Affiliations:** 1 Department of Visceral, Thoracic and Vascular Surgery, Faculty of Medicine and University Hospital Carl Gustav Carus, Technische Universität Dresden, Dresden, Germany; 2 Centre for Tactile Internet with Human-in-the-Loop (CeTI), Technische Universität Dresden, Dresden, Germany; 3 National Center for Tumor Diseases (NCT), Dresden, Germany

**Keywords:** Minimally invasive surgery, Dehydration, Laparoscopic skill analysis

## Abstract

**Introduction:**

During laparoscopic surgery, surgeons may experience prolonged periods without fluid intake, which might impact surgical performance, yet there are no objective data investigating this issue. Therefore, the aim of this study was to elucidate the effect of prolonged dehydration on laparoscopic surgical performance and tissue handling.

**Methods:**

A total of 51 laparoscopic novices participated in a single-center, open-label, prospective randomized cross-over trial. All participants were trained to proficiency using a standardized laparoscopic training curriculum. Afterward, all participants performed four different laparoscopic tasks twice, once after 6 h without liquid intake (dehydrated group) and once without any restrictions (control group). Primary endpoints were tissue handling defined by force exertion, task time, and error rate. The real hydration status was assessed by biological parameters, like heart rate, blood pressure, and blood gas analysis.

**Results:**

51 laparoscopic novices finished the curriculum and completed the tasks under both hydrated and dehydrated conditions. There were no significant differences in mean non-zero and peak force between the groups. However, dehydrated participants showed significantly slower task times in the Peg transfer task (hydrated: 139.2 s vs. dehydrated: 147.9 s, *p* = 0.034) and more errors regarding the precision in the laparoscopic suture and knot task (hydrated: 15.7% accuracy rate vs. dehydrated: 41.2% accuracy rate, *p* < 0.001).

**Conclusion:**

Prolonged periods of dehydration do not appear to have a substantial effect on the fundamental tissue handling skills in terms of force exertion among surgical novices. Nevertheless, the observed impact on speed and precision warrants attention.

**Supplementary Information:**

The online version contains supplementary material available at 10.1007/s00464-023-10644-3.

Laparoscopic surgery is a widely used technique that offers numerous advantages over traditional open surgery, including smaller incisions, reduced pain, and faster recovery. However, the intricate nature of minimally invasive surgery requires exceptional manual dexterity, precise hand–eye coordination, and cognitive focus [1]. Any factor that impairs these essential skills can potentially compromise the safety and efficacy of laparoscopic procedures [2]. A possible influencing factor, due to the long operation times, may be the surgeons’ refraining from fluid intake [3].

Dehydration is defined as a pathophysiological condition resulting from an inadequate intake of fluids or excessive fluid loss from the body [4]. With respect to the impact on the extracellular compartment, hypotonic, isotonic, and hypertonic forms of dehydration can be distinguished [5].

A growing body of evidence suggest that dehydration can significantly affect cognitive function, mood, and physical performance not only in elderly patients but also in medical personnel [6–9]. El-Sharkawy et al. investigated the prevalence of dehydration among nurses and doctors on-call and showed that dehydration commonly occurs in this population and results in impaired cognitive functions [10]. In line with this, Adan et al. found that cognitive performance was impaired in individuals with dehydration, highlighting the potential impact on decision-making and problem-solving abilities [6]. Additionally, Ganio et al. reported that even mild dehydration adversely affected cognitive performance and mood in both men and women [11].

Hwang et al. reported that the majority of surgical trainees (79%) experienced dehydration within 6 h of surgery. Moreover, 70% of the participants believed that dehydration regularly affects their performance [3]. Palejwala et al. demonstrated that hot operating rooms induce dehydration and slightly impair manual dexterity while improving alertness [12].

These findings raise concerns about the potential consequences of dehydration on the skills required for successful laparoscopic surgery. Due to a lack of evidence concerning the effects of dehydration on surgical performance, this study investigates the impact of dehydration on laparoscopic skills.

## Material and methods

This article was written in accordance with the CONSORT (Consolidated Standards of Reporting Trials) statement [13]. Due to exploratory nature of our research and the lack of available data for the impact of dehydration on surgical skills, we decided not to perform a power calculation, as the results would have been vague. The inclusion criteria required age over 18 years, informed consent to participate in the study, participation in our modified FLS training and subsequent achievement of a predefined proficiency level. Previous participation in other laparoscopy training, lack of consent or medical conditions that did not allow dehydration were criteria for exclusion of subjects.

The study was conducted as a single-center, prospective, open-label, randomized, crossover trial. Ethical approval for the study was obtained from the ethics committee of the Technische Universität Dresden (EK416092015), and written informed consent was obtained from all participants. The trial is registered at the German Clinical Trial Registry (DRKS) under DRKS00032691.

### Participants

This study was conducted at the University Hospital Dresden between October 2022 and June 2023.The study cohort consisted of fifty-one medical students without previous participation in a laparoscopic training course. All participants were trained according to a modified *Fundamentals of Laparoscopic Surgery* (FLS) training curriculum until they achieved a predefined threshold level of proficiency, which was described in detail previously [14].

After reaching proficiency, all participants had to perform four tasks in a standardized order (Peg transfer, circle cutting, balloon resection, surgical knot) twice, once being dehydrated and once being hydrated. The order of the interventions was randomized using a randomizing software to avoid a potential learning effect.

### Dehydration measurement (intervention)

All participants were instructed to abstain from fluid intake for a period of 6 h preceding the examination, typically conducted in the late afternoon (between 4 and 8 p.m.). The dehydration time of 6 h was chosen based on the results of a study conducted by Hwang et al. [3]. However, for the exclusive investigation of dehydration effects, participants were allowed to consume solid food, excluding liquid-based items, such as soups or shakes, prior to the dehydration period. Additionally, participants were permitted to consume fluids as usual until the initiation of the 6 h dehydration period.

### Hydration measurement (control)

For this measurement, all participants were given the liberty to consume both food and fluids according to their normal routine leading up to the scheduled examination.

### Measurement

The test sessions took place in the laparoscopic training laboratory of the University Hospital Dresden, which provided a controlled environment devoid of any other influencing factors that could potentially introduce bias. Between both tests, a minimum wash out period of 24 h was ensured to account for possible learning effects or influences from prior dehydration. Participants were allowed to perform their daily activity, but advised to not perform excessive exercise during the time of dehydration. For measurement of surgical performance, the ForceSense device (Medishield B.V., Delft, the Netherlands) was used. Therefore, the various tasks were attached on a platform to the ForceSense device within a standard laparoscopic box trainer. The ForceSense system is capable of measuring the motion of two laparoscopic instruments independently. Furthermore, the force exertion [Newton] on the task platforms can be measured.

### Endpoints—assessment of dehydration

In order to assess participants’ hydration status objectively, vital parameters, such as blood pressure, heart rate, and body temperature, were measured prior to the test. Additionally, a blood gas analysis taken from the fingertip was analyzed (including pH, hematocrit, hemoglobine, sodium, potassium, chloride, calcium, glucose, and lactate). Additionally, subjective measures related to hydration, including the level of thirst, were assessed through a comprehensive questionnaire. This questionnaire encompassed inquiries regarding liquid intake patterns, perceived level of thirst, and other relevant aspects related to hydration.

### Primary endpoint—force analyses and task completion time

Laparoscopic performance was assessed using ForceSense (MediShield B.V., Delft, The Netherlands) for measuring several force-related parameters. Among others, peak force, and mean non-zero force were evaluated during the measurements. The analyzed parameters were already described by Hardon et al. and listed in the following [15]:

- *Maximal force *(*N*) The highest absolute force exerted by the instruments on the task platform during the task.
- *Mean non-zero force *(*N*) Mean absolute force exerted by the instruments on the task platform during periods when force is not zero.
- *Task completion time* (s) Time measured from the beginning of the task until task completion.

### Secondary endpoint

#### Motion analyses: mean speed, path length, and volume of motion

Apart from force and time-based surgical assessment, the ForceSense system is further capable of motion analysis. Therefore, mean speed, path length, and volume of motion and mean speed were measured for both hands individually under both test conditions. The measured parameters were defined as followed [15]:

- *Path length* (mm) Total distance traveled by the tip of the right and left instrument together during the task.
- *Motion volume* (mm^3^) Volume calculated, respectively, by the widest motion of the instruments of interest on the *x*-, *y*-, and *z*-axes.
- *Mean speed* (cm/s) Average instrument speed per second.

#### Surgical errors

Significant errors were specifically defined for each task and recorded for each measurement (Supplementary Material Table 1). Error definition was similar as described previously: Peg transfer (triangle lost), circle cutting (cutting outside the line), and Balloon resection (Balloon perforation), indicating unsuccessful task completion. For the surgical knot task, three distinct error criteria were defined: imprecise suturing, incomplete suture approximation, and faulty knot tightness [16]. One individual blindly recorded error occurrences for all tasks.

#### Psychological workload

The evaluation of psychological workload was conducted using the NASA Task Load Index (NASA-TLX) [17]. This index enables participants to assess their perceived workload through various rating options. The scoring system encompasses a comprehensive evaluation where participants rank different domains (such as mental demand, physical demand, temporal demand, performance, effort, and frustration level) in relation to each other. This ranking is performed once for all tasks performed under hydration and once for all tasks performed under dehydration. Subsequently, participants assign ratings to these six domains on a scale ranging from 0 to 100, thereby quantifying their perceived mental workload following each task. These individual domain ratings are then multiplied by an overarching scale factor and aggregated to yield a summary score.

### Statistical analysis

Statistical analysis was conducted using SPSS version 28 (IMB Corp, Armonk NY, USA). The normality of continuous data was tested using the Kolmogorov–Smirnov test as well as by inspecting the frequency distributions. The participant characteristics are displayed as mean values and corresponding standard deviations (SD) for continuous variables or as distributions of frequencies. Depending on the data characteristics, the appropriate statistical test was utilized (paired student’s *t* test, McNemar’s test, Wilcoxon rank test) to compare between conditions. No missing data occurred for the primary analysis. A *p* value smaller than 0.05 was considered statistically significant.

## Results

### Basic participant characteristics

Fifty-one medical students participated in this single-center, prospective randomized cross-over trial. The participants had a median age of 24.5 years (SD = 3.4 years). All participants were students who had completed at least the second year of their medical studies. The study group comprised 20 male and 30 female participants, one participant did not specify gender. The majority of the students were right-handed (92.2%), and 8 participants had prior experience in laparoscopic surgery (15.7%) (Table 1).

**Table 1:** Basic participant characteristics

Basis participant characteristics	Values
Age, mean [years (SD)]	24.5 (3.4)
Sex, number [*n* (%)]	
Male	20 (39.2)
Female	30 (58.8)
Diverse	1 (2)
Laparoscopic experience, number [*n* (%)]	8 (15.7)
Sleep, mean (SD)	7.22 (0.9)
Physical activity per week, number (%)	
< 2 h	10 (19.6)
3–4 h	19 (37.3)
5–6 h	11 (21.6)
7–8 h	9 (17.6)
> 8 h	2 (3.9)
Smoking, number (%)	3 (5.9)

### Impact of dehydration on participants

Under dehydrated conditions, participants reported a significantly lower fluid intake throughout the same day (hydrated: 76.5% > 1 l vs. dehydrated: 19.6% > 1 l; *p* < 0.001). During the dehydration period no fluid intake was noted in the dehydration group. In line with this, participants revealed a significantly higher level of thirst at the time point of dehydrated measurement (hydrated: 0.29 vs. dehydrated: 1.47; *p* < 0.001). Apart from subjective parameters, clinical parameters also differed between both conditions. Blood gas analyses showed that hemoglobin levels (hydrated: 9.2 mmol/l vs. dehydrated: 9.4 mmol/l; *p* = 0.023) and hematocrit (hydrated: 45.5% vs. dehydrated: 46.3%; *p* = 0.018) were significantly higher under dehydration. Participants also exhibited significantly higher chloride levels under dehydrated conditions (hydrated: 108.4 mmol/l vs. dehydrated: 109.2 mmol/l; *p* = 0.006), while sodium, potassium, calcium, lactate, and glucose levels as well as the pH-value did not differ between both groups. Vital parameters like blood pressure, heart frequency, and body temperature did not differ between groups (Table 2).

**Table 2:** Comparison of blood gas analysis results between the hydrated and dehydrated group

	Hydrated	Dehydrated	*p* value
Fluid intake, number [*n* (%)]			** < 0.001**
0 l	0 (0)	9 (17.6)	
< 1.0 l	12 (23.5)	32 (62.7)	
1-2 l	33 (64.7)	8 (15.7)	
> 2 l	6 (11.8)	2 (3.9)	
Level of thirst, number [*n* (%)]			** < 0.001**
Low	36 (70.6)	3 (5.9)	
Moderate	15 (29.4)	21 (41.2)	
High	0 (0)	27 (52.9)	
Blood gas analysis, mean (SD)			
Hemoglobin [g/dl (SD)]	9.2 (1)	9.4 (1)	**0.023**
Hematocrit [% (SD)]	45.5 (4.9)	46.3 (4.9)	**0.018**
pH [(SD)]	7.4 (0.03)	7.4 (0.03)	0.406
Chloride [mmol/l (SD)]	108.4 (2.1)	109.2 (2.2)	**0.006**
Sodium [mmol/l (SD)]	144.6 (2.8)	145.4 (3.4)	0.113
Potassium [mmol/l (SD)]	4.6 (0.6)	4.7 (0.6)	0.421
Calcium [mmol/l (SD)]	1.2 (0.04)	1.2 (0.04)	0.683
Glucose [mmol/l (SD)]	5.6 (0.8)	5.4 (0.7)	0.196
Lactate [mmol/l (SD)]	1.7 (0.5)	1.8 (1.1)	0.816

Significant *p* values are highlighted in bold

### Task completion time and force analysis

Under dehydrated conditions, Peg transfer time was significantly longer compared to the hydrated control (hydrated: 139.2 s vs. dehydrated: 147.9 s; *p* = 0.034). However, this effect was not observed in the other tasks, such as circle cutting (hydrated: 214 s vs. dehydrated: 214.7 s; *p* = 0.849), Balloon resection (hydrated: 211.1 s vs. dehydrated: 200.1 s; *p* = 0.359), and surgical knot (hydrated: 308.3 vs. dehydrated: 280.4 s; *p* = 0.207) which showed no significant differences.

When analyzing the applied non-zero force during the laparoscopic tasks, no statistically significant differences were found in the Peg transfer task (0.77 N vs. 0.76 N; *p* = 0.717), in the circle cutting task (0.78 N vs. 0.82 N; *p* = 0.231), in the Balloon resection task (1.29 N vs. 1.24 N; *p* = 0.45), and surgical knot task (0.84 N vs. 0.81 N; *p* = 0.196) between the hydrated and dehydrated conditions. Similarly, concerning their maximal applied force, participants revealed no statistically significant differences regarding the Peg transfer (3.1 N vs. 3.4 N; *p* = 0.217), circle cutting (3.1 N vs. 2.95 N; *p* = 0.941), the Balloon resection (6.23 N vs. 6.69 N; *p* = 0.433),and the surgical knot task (3.85 N vs. 3.64 N; *p* = 0.483) when comparing the hydrated and the dehydrated measurements (Table 3).

**Table 3:** Comparison of task completion time and force exertion between hydrated and dehydrated groups

	Hydrated mean (SD)	Dehydrated mean (SD)	*p* value
Peg transfer, mean			
Time (s)	139.2 (27)	147.9 (36.1)	**0.034**
Peak force (N)	3.1 (1.16)	3.4 (1.6)	0.217
Mean non-zero force (N)	0.76 (0.14)	0.77 (0.17)	0.717
Circle cutting, mean (SD)			
Time (s)	214 (64.5)	214.7 (62.6)	0.849
Peak force (N)	2.97 (0.89)	2.95 (1.13)	0.941
Mean non-zero force (N)	0.82 (0.23)	0.78 (0.23)	0.231
Balloon resection, mean (SD)			
Time (s)	211.1 (94.3)	200.1 (82)	0.359
Peak force (N)	6.23 (3.87)	6.69 (4.46)	0.433
Mean non-zero force (N)	1.24 (0.45)	1.29 (0.4)	0.45
Suture and knot, mean (SD)			
Time (s)	308.3 (119.1)	280.4 (109.3)	0.207
Peak force (N)	3.85 (1.88)	3.64 (1.43)	0.483
Mean non-zero force (N)	0.81 (0.16)	0.84 (0.22)	0.196

Significant *p* values are highlighted in bold

### Motion analysis: mean speed, path length, and volume of motion

The motion analysis for mean speed did not show significant differences between the hydration and dehydration conditions for any of the performed exercises. Likewise, the total path length was not significantly different for the performed tasks for the conditions. The measured total volume of motion showed ambiguous results and significant differences between the left and right hand for the circle cutting and knot-tying exercise (Table 4).

**Table 4:** Comparison of laparoscopic motion performance between the hydrated and dehydrated groups

	Hydrated	Dehydrated	*p* value
	mean (SD)	mean (SD)	
Peg transfer			
Mean speed left hand (cm/s)	3.19 (0.38)	3.13 (0.37)	0.228
Mean speed right hand (cm/s)	3.1 (1.16)	3.13 (0.44)	0.084
Total path length (mm)	8094.7 (1718.6)	8416.2 (2226.6)	0.192
Volume of motion left hand (mm^3^)	6544.7 (2536.2)	6976.1 (2314)	0.186
Volume of motion right hand (mm^3^)	9418.5 (4768)	9975.4 (4706.1)	0.455
Circle cutting			
Mean speed left hand (cm/s)	2.34 (0.36)	2.3 (0.32)	0.353
Mean speed right hand (cm/s)	2.66 (0.5)	2.64 (0.56)	0.833
Total path length (mm)	3793.6 (1415.1)	9097.1 (3432.8)	0.787
Volume of motion left hand (mm^3^)	9539.9 (4984)	7165.3 (3161.9)	** < 0.001**
Volume of motion right hand (mm^3^)	9314.6 (2820.7)	9275 (3415.1)	0.983
Balloon resection			
Mean speed left hand (cm/s)	2.48 (0.46)	2.47 (0.48)	0.781
Mean speed right hand (cm/s)	2.48 (0.53)	2.51 (0.49)	0.629
Total path length (mm)	8842.4 (4772)	8274 (3316.6)	0.361
Volume of motion left hand (mm^3^)	4555.2 (2456)	5210.2 (3883.4)	0.222
Volume of motion right hand (mm^3^)	7927.2 (3490)	9401.5 (5607.1)	0.065
Suture and knot			
Mean speed left hand (cm/s)	2.41 (0.37)	2.4 (0.43)	0.965
Mean speed right hand (cm/s)	2.82 (0.5)	2.82 (0.49)	0.997
Total path length (mm)	14,276 (5788.6)	13,012.4 (5611.7)	0.229
Volume of motion left hand (mm^3^)	10,587.9 (3700.7)	10,503 (4284.6)	0.903
Volume of motion right hand (mm^3^)	13,472.3 (4884.2)	11,574.1 (3676.7)	**0.011**

Significant *p* values are highlighted in bold

### Surgical errors

Concerning Peg transfer, participants revealed no significant increase in the occurrence of errors (hydrated: 27.5% vs. dehydrated: 35.3%; *p* = 0.29). Similarly, there were also no significant differences regarding errors in the circle cutting task (hydrated: 21.6% vs. dehydrated: 23.5%; *p* = 0.152). The occurrence of errors in the Balloon resection task was comparable in both groups (hydrated: 51% vs. dehydrated 45.1%; *p* = 0.475).

Regarding the laparoscopic suture and knot task, the dehydrated group performed worse regarding the tightness (2% vs. 13.7%; *p* = 0.248) and adaption (2% vs. 11.8%; *p* = 0.132) of the knot but the difference was not significant. However, inaccurate stitching occurred significantly more frequently under dehydrated conditions (hydrated: 15.7% vs. dehydrated 41.2%; *p* < 0.001) (Table 5).

**Table 5:** Comparison of error occurrence between hydrated and dehydrated groups

	Hydrated n (%)	Dehydrated n (%)	*p* value
Peg transfer	14 (27.5)	18 (35.3)	0.29
Circle cutting			0.152
> 5 mm deviation from marked line	11 (21.6)	12 (23.5)	
> 10 mm deviation from marked line	3 (5.9)	6 (11.8)	
Balloon resection			0.475
Micro perforation	1 (2)	2 (3)	
Macro perforation	26 (51)	23 (45.1)	
Suture and knot			
Precise stitches			**0.001**
All correct	28 (54.9)	14 (27.5)	
Incorrect stitch	15 (29.4)	16 (31.4)	
Incorrect stitches	8 (15.7)	21 (41.2)	
Penrose adaption			0.132
Completely adapted	49 (96.1)	44 (86.3)	
Partly adapted	1 (2)	6 (11.8)	
Not adapted	1 (2)	1 (2)	
Knot tightness			0.248
Tight knots	49 (96.1)	44 (86.3)	
Loose knot under pressure	1 (2)	7 (13.7)	
Visible loose knot	1 (2)	0 (0)	

Significant *p* values are highlighted in bold

### Psychological workload

The NASA-TLX was used to assess the psychological workload. The overall score was compared for each exercise during the hydrated and dehydrated measure. There were no significant differences observable in the overall scores (Table 6).

**Table 6:** Comparison of NASA-TLX scores between hydrated and dehydrated groups

Task	Hydrated mean (SD)	Dehydrated mean (SD)	*p* value
Peg transfer	50.3 (14.9)	51.2 (14.1)	0.533
Circle cutting	52.5 (13.2)	52.9 (12.7)	0.807
Balloon resection	53.4 (15.8)	53.1 (17.8)	0.996
Suture and knot	60.4 (15.9)	57.8 (16.1)	0.247

## Discussion

Laparoscopic surgeries are well-established approaches and have become to some extent standard of care even for complex oncological procedures. Inherently, with the increasing complexity of laparoscopic procedures, the procedural time increases resulting in prolonged times for surgeons without food or fluid intake. According to a survey among surgeons, over 90% of surgeons have felt dehydrated in the OR. Around 70% stated dehydration to be a significant problem among surgeons and almost the same amount of surgeons thought that dehydration might affect their performance [3]. Especially novice surgeons need longer to perform tasks and might therefore be susceptible to suffer from dehydration. This state of dehydration could deteriorate surgeons’ well-being and is a risk to patient safety [11]. However, evidence concerning the impact of dehydration on laparoscopic performance is still lacking. Therefore, this trial aimed to evaluate the effects of dehydration on the laparoscopic performance.

In this study, dehydration was induced by the restriction of fluid intake for 6 h. This time period was chosen because it represents a realistic duration of complex surgeries and results from Hwang et al. who indicated that nearly 50% of surgeons feel dehydrated after 6 h [3]. Accordingly, participants in the present trial reported a significantly higher level of thirst under dehydrated conditions. Additionally, blood gas analysis revealed significant differences for the hematocrit and the hemoglobin levels which is a common sign for dehydration [4, 18]. These findings prove the successful induction of dehydration within our intervention group.

In summary, our study revealed that a period of 6 h of dehydration did not have a significant impact on the laparoscopic performance of surgical novices. To explore potential effects of dehydration, we employed a comprehensive set of endpoints. Despite incorporating quantitative assessments, our investigation did not find any systemic effects attributable to dehydration. Consequently, we cannot assert a direct correlation between the degree of dehydration and a decline in performance. Hence, a simplified time-dependent evaluation of dehydration is insufficient to assume performance impairment due to reduced liquid intake. Particularly in this context, we must consider that the symptoms described in the study by Hwang et al. may be attributed to a plethora of potential influencing factors rather than solely the duration of dehydration [3].

Also previous studies concerning the effect of dehydration on cognitive and motoric performance have been contradictory. While Palejwala et al. revealed that hot operating rooms induce dehydration resulting in a slight impairment of manual dexterity [12], other studies showed that dehydration is rather affecting cognitive functions [6, 11]. However, Palejwala et al. showed that dehydration improved alertness, while [12] Ganio et al. and Adan et al. indicated that dehydration could impair cognitive functions [6, 11]. Regarding the assessment of participants’ psychological workload, no significant effects of dehydration were observed in this study.

Finally, we have to admit that we cannot definitively dismiss a potential connection between dehydration and laparoscopic performance. However, it is likely that this relationship is more intricate than simply assuming that 6-h periods of dehydration invariably lead to poorer laparoscopic performance. Consequently, additional research is needed, taking into account various factors that influence hydration status, in order to comprehensively evaluate the effects of dehydration in the operating room.

## Strengths and limitations

Physiological adaptation to dehydration varies individually and might impact participants differently. Participants were advised to not perform exhaustive physical exercise during the period of dehydration. Nevertheless students were not monitored during this time. Also, the dehydration period of 6 h could have been too short to induce significant effects in the participants. The quality of data indicating a sufficient time span to induce dehydration is sparse, therefore we chose the dehydration period of 6 h based on results of a survey by Hwang et al. [3] Nevertheless, the results of our blood gas analysis and the subjective feeling of thirst indicated a sufficient time span of dehydration.

Our trial included only surgical novices because they are more prone to influencing factors. Moreover, the lack of previous laparoscopic experience and the standardized proficiency-based training with subsequent testing ensured high comparability between the participants. Therefore, our findings might be relevant, especially for the very vulnerable group of young residents with little experience in minimally invasive surgery. Including more experienced surgeons might be more realistic and closer to clinical reality but the trial itself could be prone to the impact of varying experience and training.

Despite the fact that no significant differences are associated with the analysis of force exertion, the quality of these endpoints must be pointed out. The simplified representation of laparoscopic performance by reproducing the duration of individual tasks or analysis of major errors such as dropped Peg does not reflect the complexity of the laparoscopic surgery. Precisely the tissue handling and the efficiency of the motions are important parameters that must be used as relevant endpoints in performance analyses. Therefore, by choosing these endpoints, we present a complex and realistic analysis.

## Conclusion

Dehydration could lead to less precise suturing and longer task durations in surgical novices, but our results do not indicate a general deterioration of laparoscopic skills under dehydration. In general, based on our results, most laparoscopic skills of surgical novices do not seem to deteriorate significantly under dehydration, so in this regard, operative safety is mostly not compromised even during prolonged operations. By elucidating the influence of dehydration on surgical performance, this study provides valuable insights that can contribute to the development of evidence-based practices and interventions to optimize surgical well-being.

### Supplementary Information

Below is the link to the electronic supplementary material.

Supplementary file1 (DOCX 16 KB)
