# Supplementary material for: Impact of dehydration on laparoscopic performance: a prospective, open-label, randomized cross-over trial
Source: Surg Endosc. 2023 Dec 26;38(3):1390–7. doi: 10.1007/s00464-023-10644-3 (PMC10881765; doi:10.1007/s00464-023-10644-3)
Supplement: Supplementary file 1 — Supplementary file1 (DOCX 16 KB) [file 464_2023_10644_MOESM1_ESM.docx]

**Supplementary Material Table 1:** Error classification for each task. Significant errors were specifically defined for each task and recorded according to the following system which was described previously by Bechtolsheim et al. (Bechtolsheim et al., 2022).

| **Task** | **Points** | **Error definition** |
| --- | --- | --- |
| Peg | 0 | no triangle dropped |
|  | 1 | per dropped triangle |
| Circle cutting | 0 | 0-5mm cut out of the margin |
|  | 1 | for each cut >5-10mm out of the margin |
|  | 2 | for each cut >10mm out of the margin |
| Balloon resection | 0 | no perforation |
|  | 1 | micro perforation- water leaks only under applied pressure |
|  | 2 | macro perforation- water leaks without applied pressure |
| Suture-precision | 0 | suture through both points on penrose |
|  | 1 | suture through one point on penrose |
|  | 2 | suture through no point on penrose |
| Suture-adaption | 0 | both sides of penrose touch |
|  | 1 | both sides of penrose adapt but do not touch |
|  | 2 | no adaption |
| Suture-tightness | 0 | knot tight under manipulation |
|  | 1 | knot visually tight but loosens under manipulation |
|  | 2 | knot visually loose |
